# Effects of hyperoxia on ^18^F-fluoro-misonidazole brain uptake and tissue oxygen tension following middle cerebral artery occlusion in rodents: Pilot studies

**DOI:** 10.1371/journal.pone.0187087

**Published:** 2017-11-01

**Authors:** Tim D. Fryer, Sohail Ejaz, Ulf Jensen-Kondering, David J. Williamson, Sergey Sitnikov, Stephen J. Sawiak, Franklin I. Aigbirhio, Young T. Hong, Jean-Claude Baron

**Affiliations:** 1 Wolfson Brain Imaging Centre, Department of Clinical Neurosciences, University of Cambridge, Cambridge, United Kingdom; 2 Stroke Research Group, Department of Clinical Neurosciences, University of Cambridge, Cambridge, United Kingdom; 3 Department of Radiology and Neuroradiology, University Medical Center Schleswig-Holstein, Kiel, Germany; 4 Department of Neurology, Hôpital Sainte-Anne Hospital, Université Paris Descartes, INSERM U894, Paris, France; Fraunhofer Research Institution of Marine Biotechnology, GERMANY

## Abstract

**Purpose:**

Mapping brain hypoxia is a major goal for stroke diagnosis, pathophysiology and treatment monitoring. ^18^F-fluoro-misonidazole (FMISO) positron emission tomography (PET) is the gold standard hypoxia imaging method. Normobaric hyperoxia (NBO) is a promising therapy in acute stroke. In this pilot study, we tested the straightforward hypothesis that NBO would markedly reduce FMISO uptake in ischemic brain in Wistar and spontaneously hypertensive rats (SHRs), two rat strains with distinct vulnerability to brain ischemia, mimicking clinical heterogeneity.

**Methods:**

Thirteen adult male rats were randomized to distal middle cerebral artery occlusion under either 30% O_2_ or 100% O_2_. FMISO was administered intravenously and PET data acquired dynamically for 3hrs, after which magnetic resonance imaging (MRI) and tetrazolium chloride (TTC) staining were carried out to map the ischemic lesion. Both FMISO tissue uptake at 2-3hrs and FMISO kinetic rate constants, determined based on previously published kinetic modelling, were obtained for the hypoxic area. In a separate group (n = 9), tissue oxygen partial pressure (P_t_O_2_) was measured in the ischemic tissue during both control and NBO conditions.

**Results:**

As expected, the FMISO PET, MRI and TTC lesion volumes were much larger in SHRs than Wistar rats in both the control and NBO conditions. NBO did not appear to substantially reduce FMISO lesion size, nor affect the FMISO kinetic rate constants in either strain. Likewise, MRI and TTC lesion volumes were unaffected. The parallel study showed the expected increases in ischemic cortex P_t_O_2_ under NBO, although these were small in some SHRs with very low baseline P_t_O_2_.

**Conclusions:**

Despite small samples, the apparent lack of marked effects of NBO on FMISO uptake suggests that in permanent ischemia the cellular mechanisms underlying FMISO trapping in hypoxic cells may be disjointed from P_t_O_2_. Better understanding of FMISO trapping processes will be important for future applications of FMISO imaging.

## Introduction

Ischemic stroke is characterized by focal severe brain hypoxia. In turn, tissue hypoxia triggers the cellular and molecular events that lead to infarction of the ‘ischemic penumbra’ unless perfusion—and hence oxygen delivery—is rapidly restored [1–4]. Accordingly, mapping brain hypoxia is a major goal for stroke diagnosis but also to investigate stroke pathophysiology and to monitor the effects of therapeutic interventions. ^18^F-fluoro-misonidazole (FMISO) positron emission tomography (PET) is the gold standard *in vivo* hypoxia imaging method [5] and has been extensively used for stroke research in the last two decades both in rodents [6–14] and acute/subacute stroke patients [15–24]. Following IV bolus administration, plasma FMISO passively diffuses into tissues (including the brain) and washes out over time from normoxic tissues where no retention process occurs; however, in severely hypoxic tissues, FMISO is reduced to a free radical form by reductases active only in viable cells, and the reduced form is irreversibly trapped by covalent binding to intracellular macromolecules (probably large, complex proteins or DNA) according to an incompletely understood mechanism [5]. In other words, the uptake of FMISO and other misonidazole derivatives depends on the presence of both severe tissue hypoxia and cell viability. Importantly, FMISO retention in various tissues has been shown to be highly sensitive to tissue oxygen tension in a non-linear way, mildly increasing with moderate degrees of hypoxia and markedly so at severe hypoxia [25] [26]. With respect to the brain, it is also highly sensitive to perfusion, and hence oxygen delivery, in rodent models of cerebral ischemia [14, 27, 28] as well as in human stroke [15].

Consistent with these notions, rodent studies [10] have documented that while marked tracer retention occurs in hypoxic tissue when FMISO is administered early after permanent middle cerebral artery occlusion (MCAo), no tracer retention occurs if it is administered after release of the occlusion—interpreted as due to disappearance of hypoxia after reperfusion -, or 48hrs after permanent occlusion—as FMISO uptake can only occur in viable cells.

Normobaric hyperoxia (NBO, to be referred to as ‘hyperoxia’ in what follows) has been shown to improve tissue oxygenation in acute stroke. Hyperoxia indeed increases tissue oxygen partial pressure (P_t_O_2_) in the setting of acute MCAo in rodents, and reduces infarct size when administered early after temporary MCAo (see [29–31] for review).

In the present pilot study, we carried out a series of experiments to test, for the first time to our knowledge, the effects of normobaric hyperoxia on FMISO brain uptake and pharmacokinetics in a rodent MCAo model. Given that FMISO gets irreversibly trapped in hypoxic but viable tissue [5], we predicted that hyperoxia started simultaneously with FMISO administration would markedly reduce FMISO brain uptake. To test this hypothesis, we assessed not only brain tracer retention at the standard 2-3hrs post-administration time, but also FMISO kinetic rate constants, specifically the irreversible trapping constant thought to reflect tissue hypoxia, by dynamically acquiring FMISO brain data since administration time and applying our previously reported quantitative kinetic model [32]. In addition, to assess the effects of hyperoxia on FMISO uptake in distinct tissue situations, we used both Wistar rats and their spontaneously hypertensive counterparts (SHRs), whose tissue vulnerability to focal cerebral ischemia, namely rate of demise of the severely hypoxic but viable tissue (i.e., the penumbra) [33–35] and final infarct volume [33, 36–40], widely differ as a result of underlying differences in functionality and structure of the pial vascular tree [39, 41–45], in turn mimicking clinical heterogeneity. In parallel to testing the effects of hyperoxia on FMISO uptake, and to inform the findings thereof, we also assessed its effects on tissue oxygen tension in both ischemic and non-ischemic cortex in a different group of animals. Given the pilot nature of this study and the straightforward hypothesis based on indirect but strong evidence, only small subject samples were used throughout, amenable to descriptive statistics only.

## Materials and methods

### Overview

All procedures were in accordance with the ethical standards of the institution at which the studies were conducted. The study was approved by the University of Cambridge Ethical Review Panel. In accordance with the legislation of UK Animals Scientific Procedures Act 1986, the Ethical Review Board required that the study be designed to keep the number of animals used to a minimum, yet sufficient to obtain meaningful results. All subjects that participated in the study will be reported below.

The main aim of our study was to assess the effects of hyperoxia, as compared to the control condition, on FMISO trapping in the affected hemisphere. Secondary objectives were to assess the effects of hyperoxia on i) FMISO kinetic constants, determined using kinetic modelling; and ii) MR and TTC lesion volume, obtained after completion of the FMISO PET study, around 3hrs after MCAo. Based on the literature, we did not expect major effects of hyperoxia on the MR and TTC lesion volumes after permanent MCAo of >3hrs duration [31]. As already mentioned, we also carried out a parallel study on the effects of hyperoxia on brain tissue P_t_O_2_ to inform the FMISO findings, expecting a significant improvement in ischemic tissue P_t_O_2_ based on the literature (see above); these experiments were carried out on different rats from the FMISO study because it was not feasible to scan the subjects while the PO_2_ probe was inserted in the brain.

### Anesthesia

Experiments were performed in freely breathing animals. Anesthesia was induced with 4% isoflurane administered via a nose cone in a 0.3 l/min O_2_ and 0.7 l/min N_2_O mix and maintained with 2% isoflurane during surgical procedures, as per previously published protocols [31, 46]. Body temperature of the animals was monitored with a rectal probe and maintained at 37.0 ± 0.5°C using a heated pad throughout all surgical procedures. Blood oxygen saturation and heart beat were continuously monitored using a pulse-oximeter and remained within normal ranges throughout. In the hyperoxia group, rats were switched to pure oxygen (1L/min) within 5mins following MCAo (see below). This hyperoxia regimen is standard for testing the effects of normobaric oxygen therapy in rodents (see [30])

### MCAo

Distal left permanent MCAo was performed as detailed previously [47–49]. In brief, the left common carotid artery (CCA) was isolated through a ventral midline incision on the neck and a loose ligature of 4–0 silk suture was placed around it. With the rat positioned onto its right flank, a 2.5cm skin incision perpendicular to and bisecting a line between the lateral canthus of the right eye and the external auditory canal was made, and the underlying temporalis muscle excised to reveal the base of the skull. Under direct visualization, the underlying temporalis muscle was excised and craniectomy was performed under saline irrigation to expose the left MCA through a 2‐mm burr hole drilled 2 to 3 mm rostral to the fusion of the zygomatic arch with the squamosal bone. The dura was retracted to visualize the MCA at a position where it crosses the inferior cerebral vein, which lies within the rhinal fissure. A micro-aneurysm clip (#1 Sundt AVM, Codman, Raynham, USA) was placed on the MCA proximal to the point where it crosses the inferior cerebral vein in the rhinal fissure, and then the left CCA was permanently ligated. To avoid additional procedural complications that may impact tissue outcome, including blood loss from femoral cannulation, arterial blood pressure was not measured here as it is known to be already significantly elevated (>170mmHg) by 3 months of age in SHRs [50].

### PET

Six Wistar rats and seven SHRs were randomized to undergo MCAo under either control (n = 3 and 4 per strain, respectively) or hyperoxia (n = 3 per strain) conditions. These sample sizes were not based on a power calculation given the absence of literature data on expected effect size (i.e., % reduction in FMISO uptake from hyperoxia). They were considered large enough to test our straightforward hypothesis that hyperoxia would markedly reduce FMISO uptake in ischemic regions (see Introduction), also taking into consideration the aforementioned ethical requirements.

Immediately after clip placement on the MCA, the animal was positioned in the PET scanner (microPET P4, Concorde Microsystems, Knoxville, USA) with the brain located centrally in the field of view. Under the oxygen condition of the scan (control or hyperoxia), within 5mins of MCAo FMISO (77 ± 9 MBq) was administered in the tail vein over 30secs [10], with FMISO PET acquisition starting simultaneously with the injection. Dynamic PET data were acquired for 3 hours. The energy and coincidence windows were 350-650keV and 6nsec, respectively, and the voxel size 0.5x0.5x0.5mm. Venous blood samples were taken at 120, 150 and 180 mins post-injection, and plasma radioactivity concentration measured in a well counter to provide values for least squares scaling of a pre-existing arterial plasma input function [32]. Transmission scanning with a rotating ^68^Ge/^68^Ga point source was conducted at the end of the FMISO scan, with geometrically-windowed coincidence mode used to provide accurate attenuation information in the presence of the residual FMISO radioactivity.

The list-mode data were binned into sinograms for the following time frames: 8 × 15s, 6 × 30s, 15 × 1min, 5 × 2min, 30 × 5min. For each sinogram, an image was reconstructed using the PROMIS 3D filtered backprojection algorithm [51], with a Hann window applied to result in a reconstructed image resolution of 2.3mm full width at half maximum (FWHM). Image reconstruction incorporated corrections for randoms, dead time, normalisation, attenuation, decay and sensitivity.

### MRI

Immediately on completion of PET scanning the animal was transported to the nearby room for magnetic resonance imaging (MRI) (4.7T Bruker BioSpec 47/40 system; Bruker BioSpin, Ettlingen, Germany). Diffusion weighted images (DWI) were acquired using an echo-planar sequence (TR/TE 3000/35ms, 35 directions b = 1000s/mm^2^, slice thickness 1.5mm, in plane resolution 0.312mm). T2-weighted MRI was then acquired using the following parameters: TR/TEeff 15078/36ms, ETL 8, NEX 1, 256 × 256 × 128 FOV 64.0 × 64.0 × 32.0 mm^3^, isotropic resolution 250 μm^3^.

### Histology

Immediately after completion of the MRI, the animal was killed with an overdose of phenobarbital and decapitated. Tetrazolium chloride (TTC) staining was then performed according to the previously described methodology [10]. The brains were isolated and cut into 2mm thick sections, which were incubated in 0.5% TTC bath at room temperature, and then scanned on a flatbed scanner.

### Image analysis

All image analyses were carried out blind to group allocation.

#### FMISO SUV images

For each animal, an FMISO standardised uptake value (SUV) map was created by averaging the images 120-180min post-injection, and multiplying this by the ratio of animal weight (kg) to injected activity (MBq). This SUV map was manually rigidly co-registered to the MR image of the same animal. For comparison of SUV maps, each individual MR image was affine-registered to a MRI brain template of a normal rat, and this transformation was applied to the co-registered SUV map to bring it to template space.

#### FMISO hypoxic ROI

Based on our hypothesis, hyperoxia should reduce the volume with significantly increased FMISO. Accordingly, the analysis followed our previously published methodology [11]. First, a region-of-interest (ROI) was defined on the MR image that encompassed the cerebral hemisphere contralateral to the MCAo, within which the mean and standard deviation (SD) of the voxel SUV values were determined. Under the assumption of normally distributed values, a statistical threshold of p<0.001 was calculated from mean + 4.26×SD. This threshold was applied to the MCAo hemisphere to delineate the hypoxic volume [11]. The mean SUV within the FMISO hypoxic ROI was also obtained in each rat.

#### FMISO kinetic rate constants

FMISO kinetic rate constants were determined within the FMISO hypoxic and contralateral hemisphere ROIs using our previously published kinetic modelling methodology [32]. To this end, a plasma-input two-compartment irreversible kinetic model was applied to the mean hypoxic volume time-activity curve (TAC) and the corresponding TAC from the contralateral hemisphere ROI [32], resulting in the estimation of two measures of FMISO trapping per region: trapping rate (k_3_) and influx rate (K_i_).

#### MRI

DWI images displayed the expected ischemic lesions in all rats (see Results), but the metallic clip for MCAo resulted in significant artefacts. Ischemic lesion volumes were therefore delineated on the T2-weighted MRI by an experimental neuroscientist with experience in stroke imaging (SE) and blinded to subject group, by manually contouring using MRIcro (www.cabiatl.com/mricro/mricro/) the volume of increased signal across the coronal slices displaying a lesion in the left MCA territory cortex.

#### TTC lesion

Two observers blinded to subject group independently determined the TTC lesion volume from the scanned TTC sections using ImageJ (https://imagej.nih.gov/ij/). As the agreement between these observers was high (R^2^ = 0.944, p<0.001), the average TTC volumes across the two observers was used henceforth.

### MCA cortex P_t_O_2_

**Fig 1:**
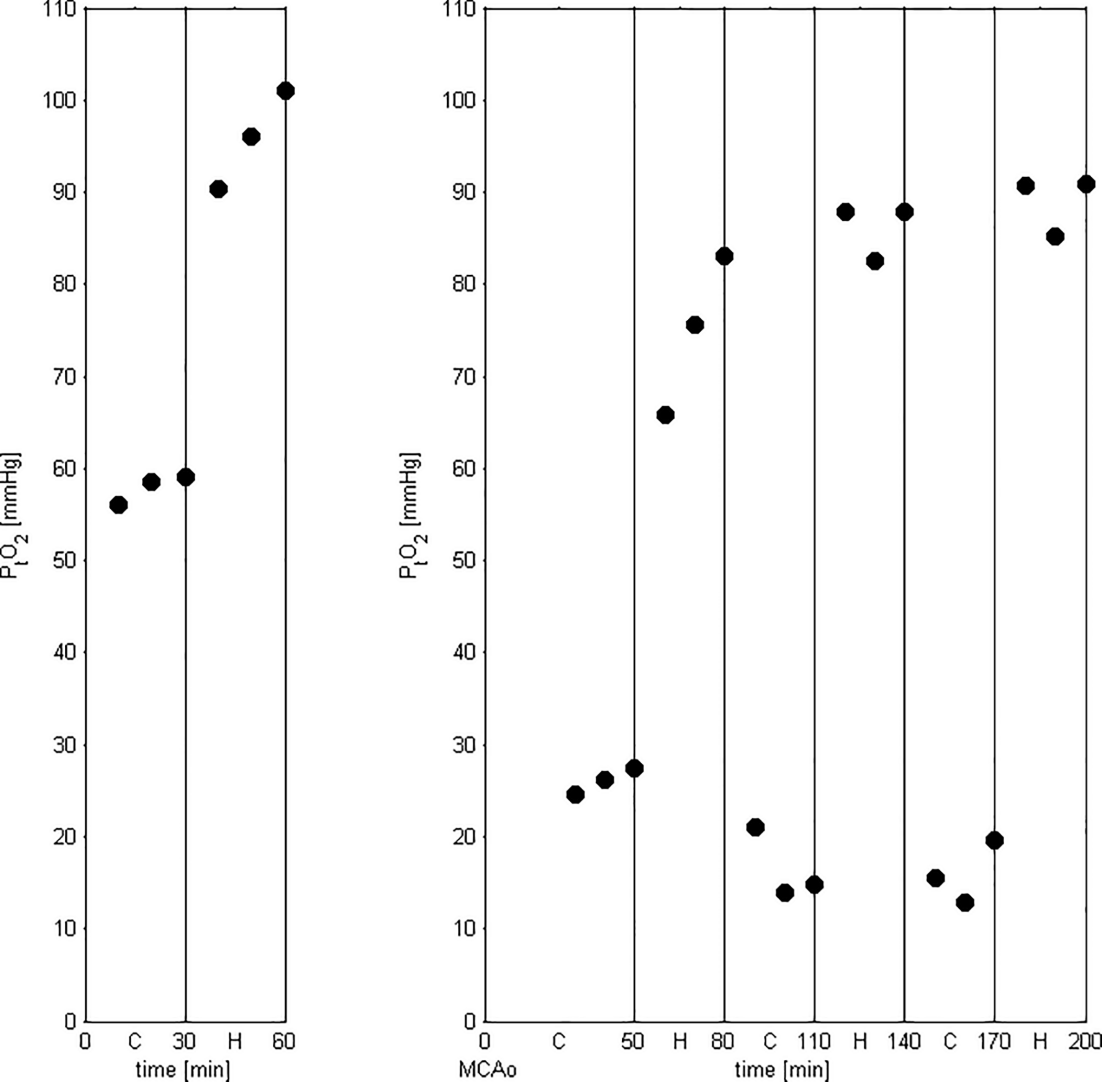
Graph showing P_t_O_2_ data from one subject to illustrate the experimental protocol. First, following an equilibration period of 30mins after probe insertion (not shown), six P_t_O_2_ measurements were obtained in the non-ischemic (right) hemisphere (3 control, 3 under hyperoxia), then MCAo and PO_2_ probe insertion were carried out on the ischemic (left) hemisphere and P_t_O_2_ was measured every 10mins (again following a 30-min equilibration period after probe insertion) across three 30min control-hyperoxia cycles (18 measurements in total). Note the rapid equilibration in P_t_O_2_ after each change of oxygen condition, i.e., within the 10mins between measurements.

The experimental protocol, including methodology for distal microclip MCAo, anesthetic regimen and physiological monitoring, was identical to that used in the PET studies above. P_t_O_2_ was measured using an O_2_ probe (Tissue Oxygenation Monitor, Oxy lab pO_2_, Oxford Optinix, Abingdon, UK) in adult male ~3 months old SHRs (n = 5; weight: ~300g) and Wistar rats (n = 4; weight: ~300g). Based on the literature and our previously published findings [48], we elected to measure P_t_O_2_ in the barrel field of the primary somatosensory cortex (S1-BF), because it is consistently part of the cortical MCA territory [52, 53] and is among the areas most severely and consistently affected after distal MCAo in SHRs [48]. To this end, under 1.5% continuous isoflurane delivery, the O_2_ probe was inserted in the S1-BF under stereotaxic conditions, using coordinates from the Paxinos atlas (bregma -2mm, lateral 6mm, depth 1mm) [54]. P_t_O_2_ was measured first in the right hemisphere starting 30mins after probe insertion to allow for resolution of acute tissue trauma as recommended. P_t_O_2_ values were then obtained every 10 mins during one 30-min control and hyperoxia cycle, allowing 10mins to elapse between the two conditions for equilibrium. Once these measurements completed, the oxygen probe was removed and left-sided distal MCAo was carried out as described above. The probe was inserted in the S1-BF of the ischemic hemisphere and measurements started 30mins after MCAo. In total, 18 measurements of P_t_O_2_ were obtained for the ischemic hemisphere, i.e., every 10 mins during three 30-min control and hyperoxia cycles, allowing 10mins to elapse between the two conditions for equilibrium, for an overall 180mins recording. The experimental design is shown in Fig 1. The P_t_O_2_ values were subsequently averaged to generate a single value for each condition and each hemisphere.

### Statistical analysis

The Anderson-Darling test [55] was used to test for normality in data distributions and differences in data (for paired data). Consequently, FMISO hypoxic lesion, MRI lesion and TTC lesion volumes were compared between-strain within-condition and within-strain between-condition using two-sample, two-tailed unequal variance t-tests. FMISO values (SUV, k_3_ and K_i_) were compared within-strain between-condition using two-sample, two-tailed unequal variance t-tests, with k_3_ and K_i_ also compared within-strain within-condition between the hypoxic and contralateral ROIs using paired, two-tailed t-tests. Note that the SUV values were not compared between hemispheres given that the hypoxic ROI was defined statistically based on contralateral hemisphere SUV (mean and SD), i.e., by definition the FMISO lesion ROI only encompassed voxels with significantly higher SUV than the mean contralateral hemisphere SUV. Correlations between FMISO hypoxic lesion, MRI lesion and TTC lesion volumes were assessed using the Pearson correlation coefficient.

P_t_O_2_ data were compared within-strain between-condition using paired, two-tailed t-tests, and between-strain for the control condition using a two-sample, two-tailed unequal variance t-test for the contralateral hemisphere and Wilcoxon rank sum for the MCAo cortex given that the SHR MCAo cortex P_t_O_2_ distribution failed the Anderson-Darling normality test (p = 0.008). Differences in P_t_O_2_ within-strain between-condition satisfied the normality test and hence these difference data were compared between-strain using two-sample, two-tailed unequal variance t-tests. Pooled P_t_O_2_ data for the MCAo cortex (i.e., by combining SHR and Wistar rats) failed normality (p = 0.008) for the difference between conditions and hence were tested between-condition using the Wilcoxon signed rank test.

Given the small samples used in the present study, statistical trends, i.e., two-tailed p values between 0.10 and 0.05, will be reported as potentially meaningful.

## Results

No animal died before the end of the experiment, and all studied animals are reported below.

**Fig 2:**
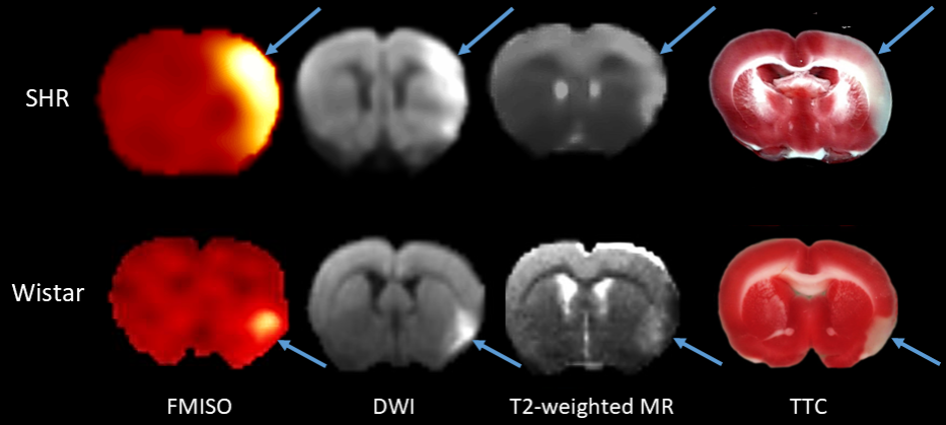
Illustrative coregistered FMISO SUV, DWI and T2-weighted MRI images for the same coronal cut in one spontaneously hypertensive rat and one Wistar rat, and approximately matched TTC coronal slice. These data illustrate the topographic congruence of the ischemic lesions (arrows) among the four imaging modalities. As TTC sections are not amenable to coregistration with PET and MR images due to differences in slice angle and thickness and potential *post-mortem* geometrical distortion, approximately same coronal level TTC sections as for the *in vivo* images are shown for illustrative purposes only.

Fig 2 shows representative coregistered FMISO SUV, DWI and T2-weighted MRI coronal sections from an SHR and a Wistar rat, illustrating conspicuous FMISO and DWI lesions, somewhat faint T2-weighted lesions, as well as the TTC lesion from approximately the same coronal section.

### Effects of hyperoxia on FMISO hypoxic ROI volumes

**Fig 3:**
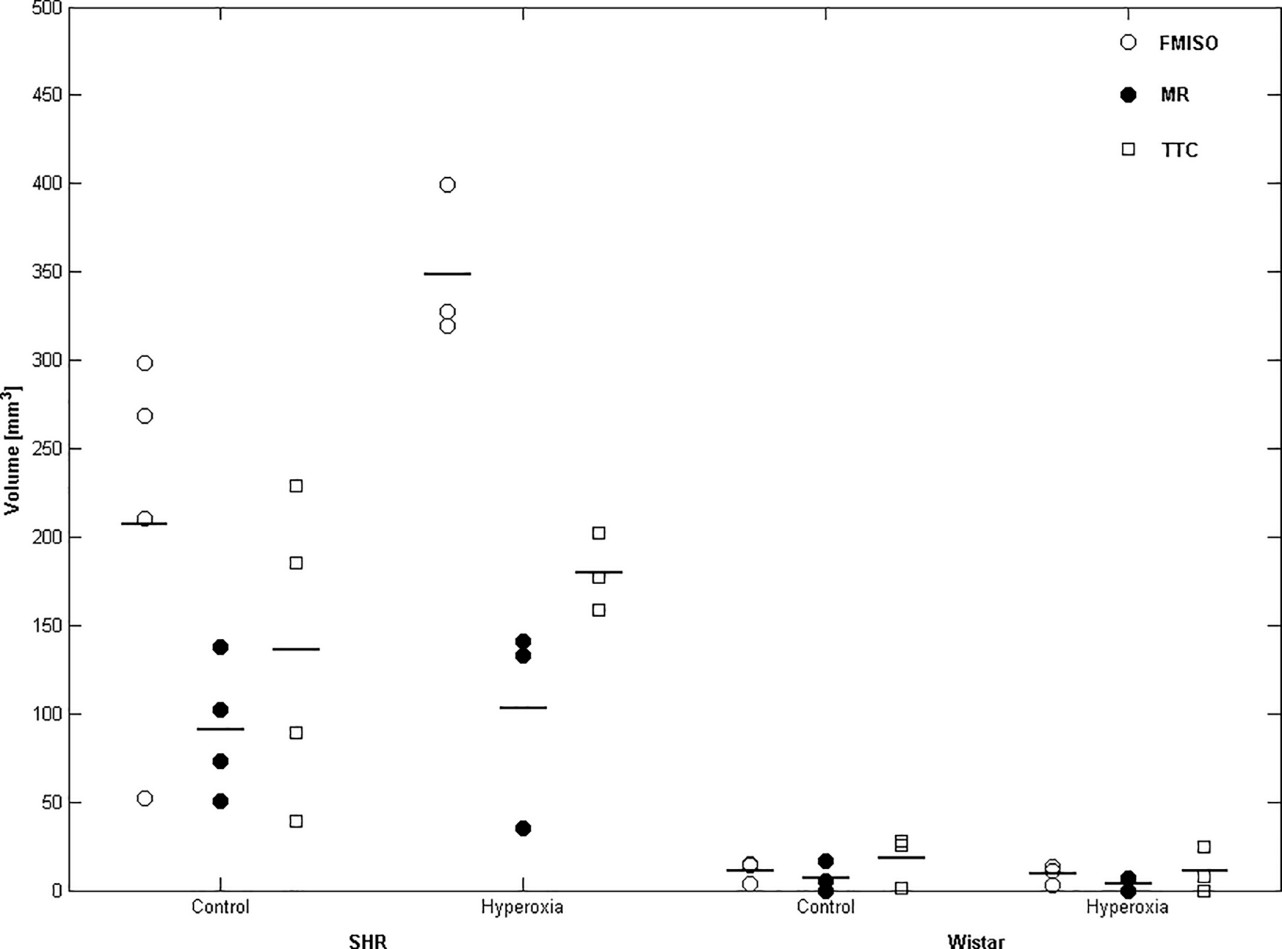
Individual and mean FMISO hypoxia, T2-weighted MRI and TTC lesion volumes for SHRs (left) and Wistar rats (right) under control and hyperoxia conditions. Horizontal lines denote the mean of each group. FMISO, MRI and TTC lesion volumes were significantly larger in SHRs than Wistar rats in both conditions (see Results section for further details), but there was no significant effect of hyperoxia in either strain. Sample sizes are n = 3–4 per group.

**Fig 4:**
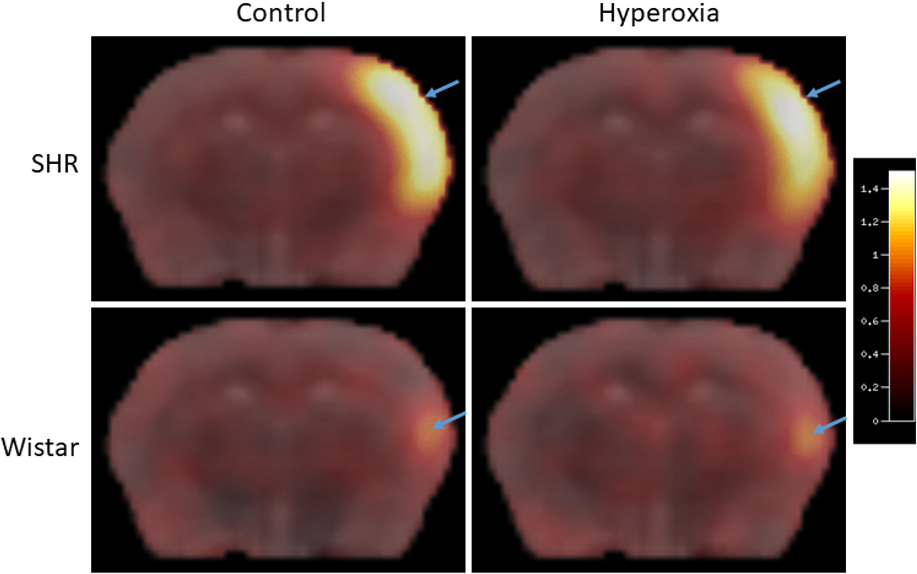
Representative FMISO SUV images overlaid on the same coronal slice of the co-registered MRI template for representative SHR (top row) and Wistar (bottom row) rats scanned under either the control (left column) or hyperoxia (right column) conditions. The pseudocolor scale on the right represents SUV units (see text for details). This illustrates the much smaller FMISO lesion induced by distal MCA occlusion in Wistar rats than SHRs, and the lack of clear effect of hyperoxia on FMISO brain uptake in either strain (see Results for further details).

During MCAo, FMISO hypoxic volumes were markedly and significantly larger in SHRs than Wistar rats in both the control (t = 3.57; p = 0.038) and hyperoxia (t = 13.34; p = 0.006) conditions; Table 1 and Fig 3. As Table 1 shows, the FMISO lesion volumes were not significantly smaller in the hyperoxia condition as compared to control in either strain, and in SHRs tended in fact to be larger (p = 0.08). Fig 4 illustrates these findings in two typical rats per strain.

**Table 1:** Mean (± SD) FMISO PET hypoxic volume and MRI and TTC lesion volumes for both rat strains and oxygen supply conditions, together with corresponding t and p values from two-sample, two-tailed, unequal variance t-tests between control and hyperoxia oxygen conditions.

Strain	Modality	Condition	Volume [mm^3^]	t (p-value)
SHR	FMISO	Control	207 ± 110	2.34 (p = 0.08)
		Hyperoxia	349 ± 44	
	MRI	Control	91 ± 38	0.31 (p = 0.78)
		Hyperoxia	103 ± 59	
	TTC	Control	136 ± 87	0.96 (p = 0.41)
		Hyperoxia	179 ± 22	
Wistar	FMISO	Control	11 ± 6	-0.35 (p = 0.74)
		Hyperoxia	9 ± 6	
	MRI	Control	7 ± 9	-0.64 (p = 0.57)
		Hyperoxia	3 ± 5	
	TTC	Control	18 ± 15	-0.66 (p = 0.54)
		Hyperoxia	11 ± 13	

FMISO, ^18^F-fluoro-misonidazole; PET, positron emission tomography; MRI, magnetic resonance imaging; TTC, tetrazolium chloride; SHR, spontaneously hypertensive rat

### Effects of hyperoxia on MRI and TTC lesion volumes

**Fig 5:**
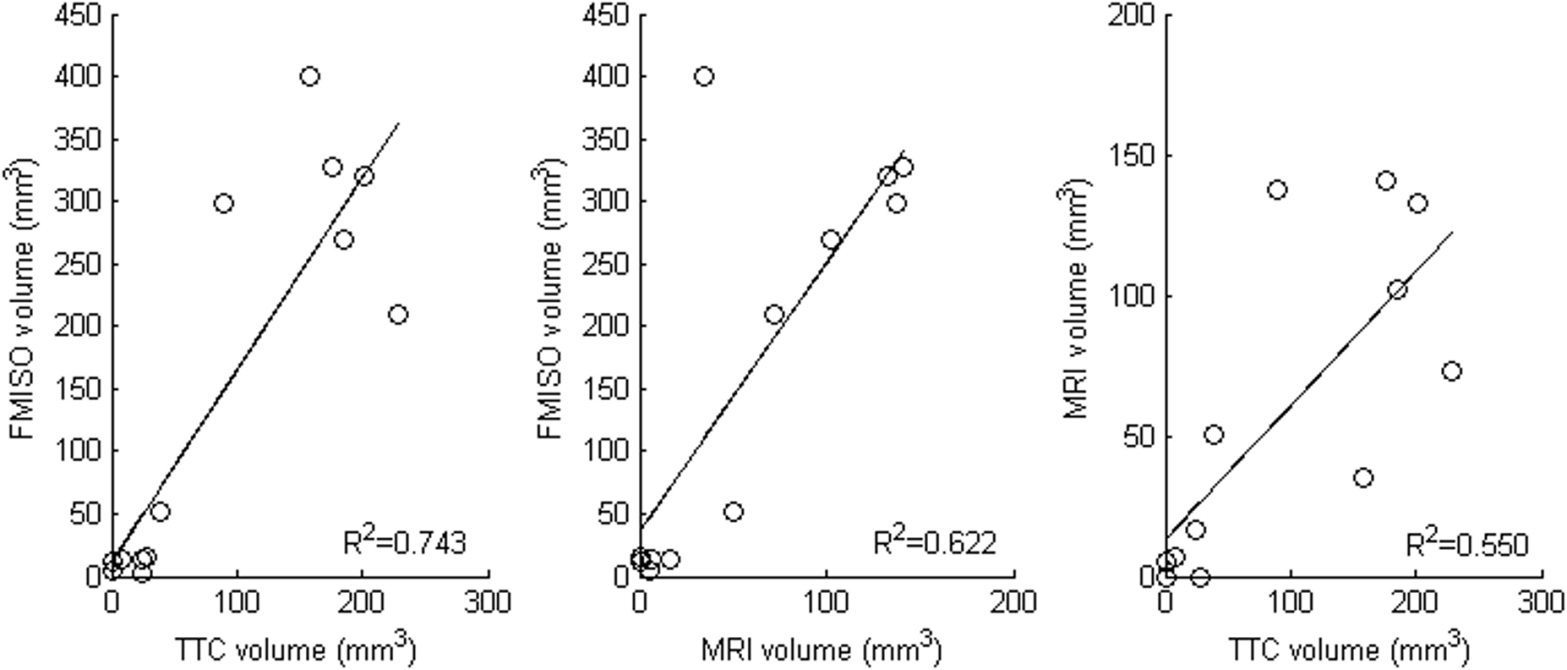
Relationships between FMISO hypoxic lesion volume, MRI lesion volume and TTC lesion volume. Each plot uses data from both SHR and Wistar rats under both the control and hyperoxia conditions (n = 13). All Pearson correlations are statistically significant (p < 0.001; R^2^ values shown next to each scatterplot).

All rats exhibited an acute hyperintense lesion on DWI, but the metallic clip placed onto the MCA consistently induced susceptibility artefacts, which precluded determination of DWI lesion volumes. Accordingly, lesion volumes were determined on the T2-weighted scans. As with the FMISO hypoxic ROI, larger MRI and TTC lesion volumes were present in SHRs as compared to Wistar rats in both control (MRI: t = 4.27, p = 0.024; TTC: t = 2.67, p = 0.08) and hyperoxia (MRI: t = 2.91, p = 0.10; TTC: t = 11.68, p = 0.001) conditions (Table 1, Fig 3). Within-strain analyses, however, revealed no significant difference between the control and hyperoxia conditions in both SHRs and Wistar rats (Table 1). Across conditions, there were robust (R^2^ = 0.55–0.74; all p<0.001) correlations among lesion volumes from FMISO PET, MRI and TTC (Fig 5).

### Effects of hyperoxia on SUV within the FMISO lesion ROI

**Fig 6:**
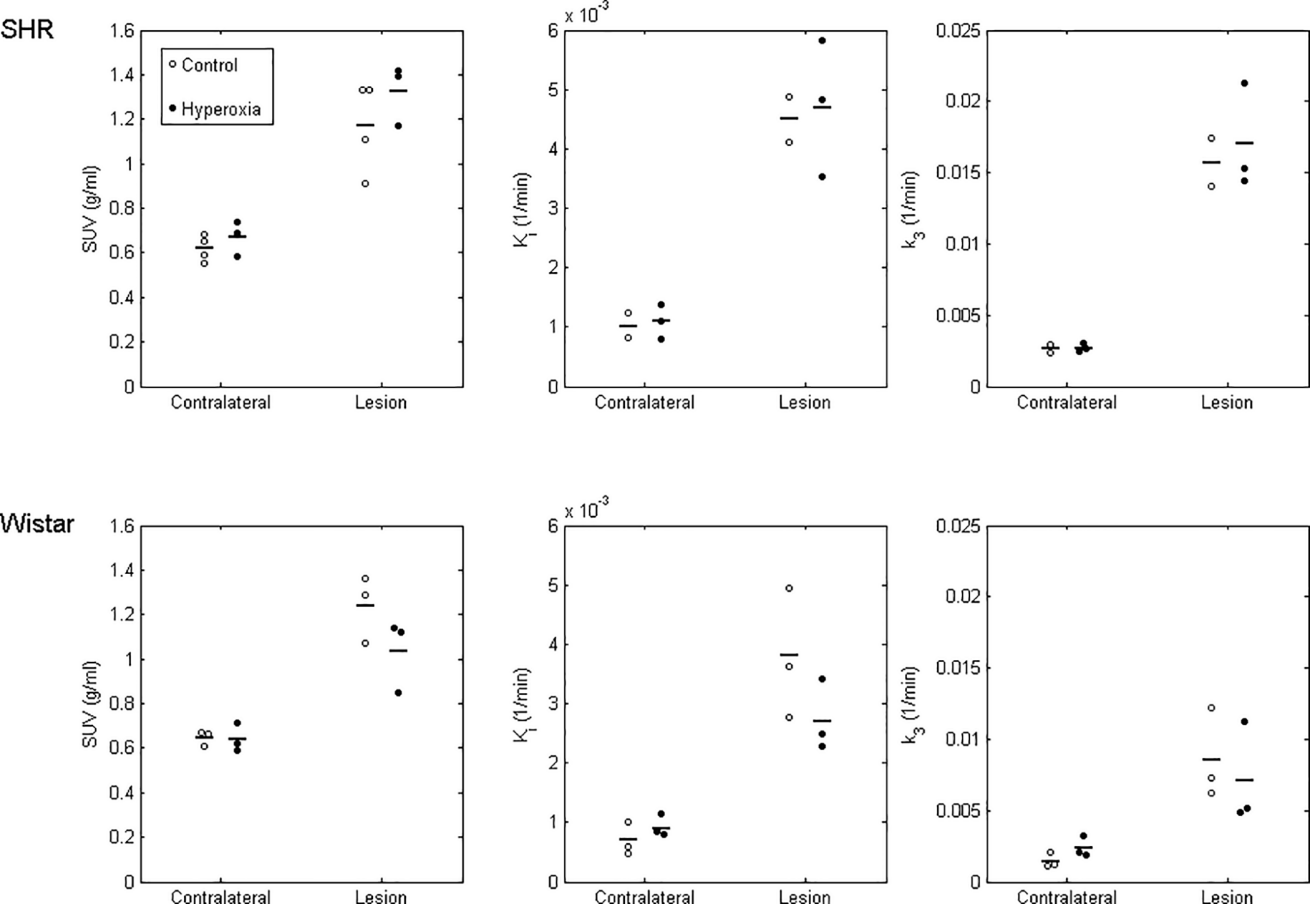
Individual and mean FMISO SUV (left column), influx rate (K_i_; middle) and trapping rate (k_3_; right) in the FMISO lesion ROI and contralateral ROI for SHR (top row) and Wistar (bottom row) rats under the control and hyperoxia conditions. Horizontal bars denote mean values. The K_i_ and k_3_ values were higher (significantly or with a strong trend) in the affected (‘lesion’) as compared to the non-ischemic (contralateral) hemisphere in both strains and both conditions (see Results section for details). However, there was no significant effect of hyperoxia on SUV, K_i_ or k_3_ in either strain.

Fig 6 shows individual and mean SUV values across both conditions and strains. FMISO lesion ROI SUV did not differ between the two conditions, either in SHRs (mean ± SD: 1.17 ± 0.20 g/ml control vs 1.33 ± 0.14 g/ml hyperoxia; t = 1.22, p = 0.28) or in Wistar rats (1.24 ± 0.15 vs 1.04 ± 0.16 g/ml; t = -1.62, p = 0.18). Also, there was no effect of hyperoxia on contralateral hemisphere SUV values in either SHR (0.62 ± 0.06 vs 0.67 ± 0.08 g/ml; t = 0.94, p = 0.40) or Wistar rats (0.65 ± 0.03 vs 0.64 ± 0.06 g/ml; t = -0.13, p = 0.91).

### Effects of hyperoxia on FMISO kinetic rate constants within the FMISO lesion ROI

**Fig 7:**
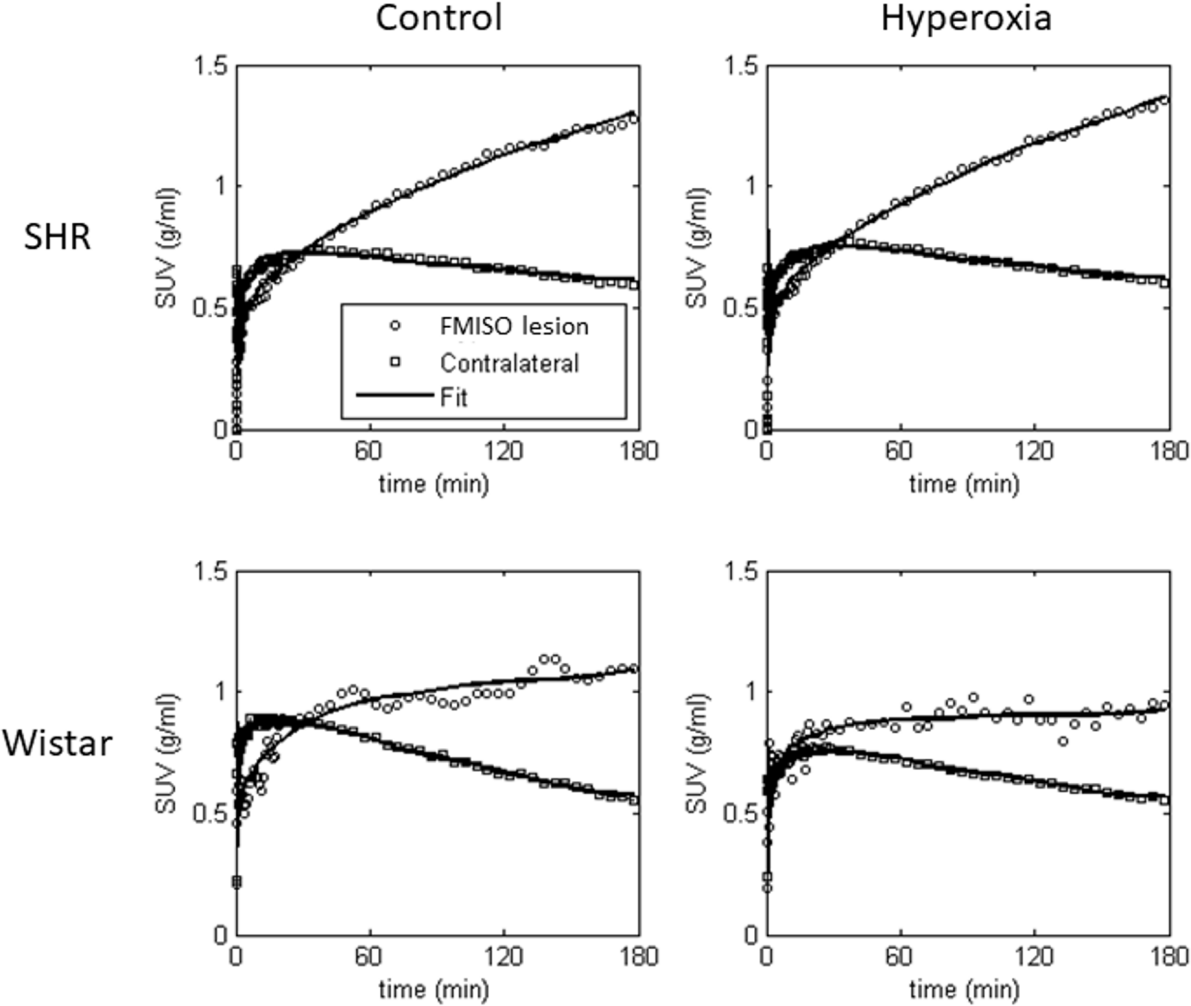
Representative FMISO time-activity curves for the FMISO hypoxic lesion ROI and contralateral ROI for the 3-hr PET scan, together with compartmental fits for SHR (top row) and Wistar (bottom row) rats under the control (left column) and hyperoxia conditions (right column).

Fig 7 shows typical examples of the compartmental model fits to the FMISO time-activity curves (TACs) for the FMISO hypoxic lesion ROI and contralateral ROI of both strains under the control and hyperoxia conditions.

As also depicted in Fig 6, FMISO influx rate (K_i_) was significantly higher in the hypoxic lesion compared to the contralateral ROI for SHR rats under both conditions (control mean ± SD: 0.0045 ± 0.0005 vs 0.0010 ± 0.0003 ml/min/ml; t = 20.41, p = 0.031; hyperoxia: 0.0047 ± 0.0011 vs 0.0011 ± 0.0003 ml/min/ml; t = 7.37, p = 0.018) and for Wistar rats under the control condition (0.0038 ± 0.0011 vs 0.0007 ± 0.0003 ml/min/ml; t = 6.44, p = 0.023), with a near significant result for Wistar rats under hyperoxia (0.0027 ± 0.0006 vs 0.0009 ± 0.0002 ml/min/ml; t = 4.19, p = 0.052). The FMISO trapping rate (k_3_) was also significantly higher in the FMISO lesion relative to its mirror ROI in SHRs under hyperoxia (0.0170 ± 0.0037 vs 0.0027 ± 0.0003 min^-1^; t = 7.14, p = 0.019) and Wistar rats in the control condition (0.0085 ± 0.0032 vs 0.0014 ± 0.0005 min^-1^; t = 4.60, p = 0.044), with similar trends in SHRs in the control condition (0.0157 ± 0.0024 vs 0.0027 ± 0.0004 min^-1^; t = 6.56, p = 0.10) and Wistar rats under hyperoxia (0.0071 ± 0.0035 vs 0.0024 ± 0.0007 min^-1^; t = 2.09, p = 0.17). However, as for SUV, hypoxic lesion K_i_ and k_3_ did not significantly differ between control and hyperoxia in either strain (|t|≤1.46, p≥0.24 in all cases).

### Effects of hyperoxia on cortical P_t_O_2_

**Fig 8:**
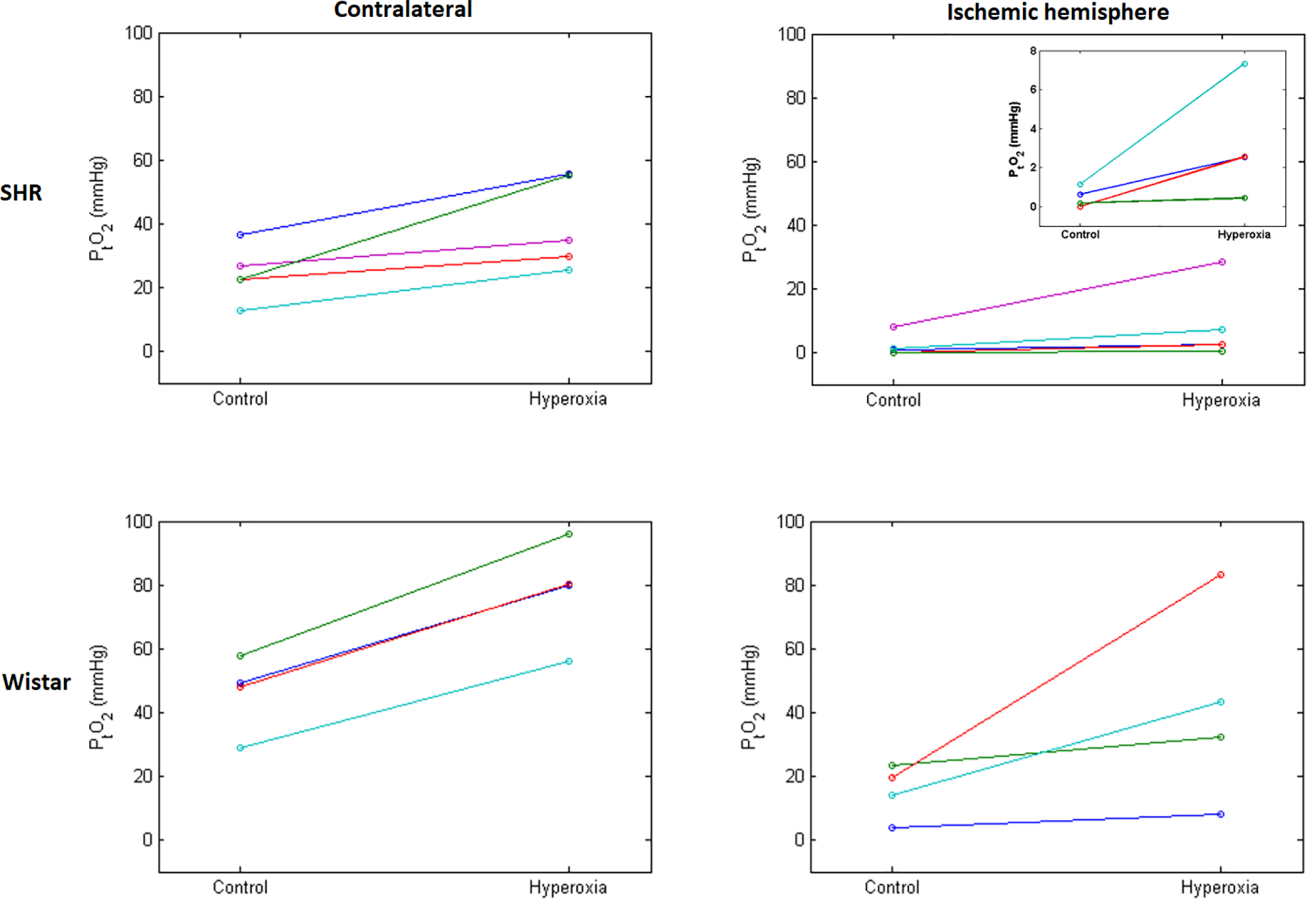
Individual cortical PO_2_ (P_t_O_2_) measured in the MCA territory in the non-ischemic hemisphere (left) and ischemic hemisphere (right) under the control and hyperoxia conditions in SHR (top) and Wistar rats (bottom; n = 5 and 4, respectively). Each line joins P_t_O_2_ values obtained under both conditions for a single rat; different colors are used for ease of illustration. The inset on the top right zooms on the four SHRs with lowest baseline P_t_O_2_ values, which are difficult to discriminate on the main graph due to the common scale used across graphs. This figure shows lower P_t_O_2_ under ischemic vs control condition in both strains, and increases in P_t_O_2_ under hyperoxia in all 9 rats (although of very small amplitude in one SHR), which was highly statistically significant with pooled data. However, due to small samples and highly variable P_t_O_2_ increases, only trends were found when strains were assessed separately. The increase in P_t_O_2_ by hyperoxia was not significantly different between the two strains in the ischemic cortex, although was significantly higher for Wistar rats in the contralateral hemisphere. Statistical tests also showed significantly higher P_t_O_2_ in the control condition in Wistar rats as compared to SHRs in both hemispheres (see Results for further details).

Fig 8 illustrates the P_t_O_2_ values in the non-ischemic and ischemic cortex in both strains and both conditions. In the non-ischemic cortex, P_t_O_2_ was significantly lower in SHRs than Wistar rats in the control condition (mean ± SD: 24.3 ± 8.6 vs 45.9 ± 12.2 mmHg; t = -3.00, p = 0.030), with significant increases in P_t_O_2_ during hyperoxia found for both the SHRs (mean increase: 15.9 mmHg; t = 3.39, p = 0.028) and Wistar rats (mean increase: 32.1 mmHg; t = 14.30, p = 0.001). The increase in P_t_O_2_ for Wistar rats was significantly greater than for SHRs (t = 3.11, p = 0.021). Increases in P_t_O_2_ had a positive correlation trend with initial P_t_O_2_ for Wistar rats (R^2^ = 0.84, p = 0.08)—i.e., the higher the baseline P_t_O_2_, the larger the increase -, but not for SHRs (R^2^ = 0.02, p = 0.84).

Ischemic cortex P_t_O_2_ was also significantly lower in SHRs than in Wistar rats in the control condition (median 0.6 vs 16.8 mmHg; p = 0.032; Wilcoxon rank sum). As shown in Fig 8, ischemic cortex P_t_O_2_ increased during hyperoxia in all 9 rats although to highly variable degrees. The increase in P_t_O_2_ was statistically significant when pooling the two strains (p = 0.004; n = 9; Wilcoxon signed rank). Due to the small samples for each strain, increases in ischemic cortex P_t_O_2_ only showed a weak trend when tested separately for SHRs (mean increase: 6.2 mmHg; t = 1.72, p = 0.16; n = 5) and Wistar rats (mean increase: 26.4 mmHg, t = 1.95, p = 0.15; n = 4). The increases in P_t_O_2_ for SHRs and Wistar rats were not significantly different (t = 1.44, p = 0.25). Increases in P_t_O_2_ were found to positively correlate with initial P_t_O_2_ for SHRs (R^2^ = 0.97, p = 0.003), but not for Wistar rats (R^2^ = 0.15, p = 0.62).

## Discussion

This pilot study is the first to assess the effects of hyperoxia on FMISO uptake during cerebral ischemia. Our experiments were designed to test a straightforward hypothesis, namely that hyperoxia initiated within minutes of onset of focal cerebral ischemia would markedly reduce FMISO uptake in the occluded MCA territory. Accordingly, only small samples and descriptive statistics were thought adequate to demonstrate this. Contrary to our expectations, however, neither the FMISO hypoxic lesion volume nor the FMISO SUVs and kinetic rate constants within this region were clearly affected in either rat strain. This included k_3_ which represents unidirectional FMISO cell trapping assumed to directly reflect tissue hypoxia [26, 32].

To test our hypothesis, we elected to use a 3hr MCA occlusion paradigm. This was necessary because 3hrs of tracer uptake are recommended to obtain reliable data from FMISO studies, due to its slow tissue kinetics and trapping in hypoxic tissue [5] (see Fig 7 for an illustration). In addition, with any radiotracer study steady-state physiological conditions are required throughout data acquisition, precluding the use of temporary MCAo with reperfusion before the 3hr time-point. Unfortunately, as of today there is no alternative brain-penetrant *in vivo* hypoxia tracer with faster kinetics or reversible trapping [5, 56].

Our hypothesis was underpinned by earlier reports that FMISO trapping strongly depends on the degree of hypoxia, be it for the liver [57] or tumors [26]. How can these unexpected results be explained? First, small samples could have caused Type 2 error, i.e., lack of statistical significance despite the presence of a difference. However–again contrary to our expectations—no discernible or consistent effect on FMISO lesion volume, SUV or kinetic rate constants was observed in either strain, and, if anything, a trend for *enlarged* FMISO lesion was present in SHRs (Table 1).

Second, the hyperoxia regimen implemented here was perhaps insufficient to substantially improve ischemic tissue oxygenation and in turn reduce FMISO uptake. However, it increased arterial PO_2_ 4-fold (from mean 121.5mmHg to mean 478.5mmHg; corresponding S_a_O_2_ values: 98.7% and 100%, respectively) [31]. Despite such marked P_a_O_2_ rises, normobaric hyperoxia is known not to result in major increases in arterial oxygen content, and hence in oxygen delivery to tissues. However, hyperbaric hyperoxia is a cumbersome procedure that would not be compatible with PET and MRI scanning. Consistent with the literature [46, 58–60], the NBO regimen applied here did however result in significant increases in ischemic cortex P_t_O_2_ across the two strains (i.e., pooled data)—although within strain these increases did not quite reach statistical significance, probably due to small samples and variable changes.

Third, P_t_O_2_ was measured in a small volume whereas we assessed FMISO uptake throughout the brain, which might account for our results. However, we purposely elected the S1-BF area for P_t_O_2_ assessments because this cortical region is well within the MCA territory [52, 53], and was previously shown to exhibit very low perfusion values after distal MCAo in SHRs [48]. Secondly, we performed a *post-hoc* analysis of FMISO SUV within the S1-BF ROI, as per our previously published methodology [31, 61]. To this end, the Paxinos stereotaxic atlas [54] was co-registered onto MRI brain templates from normal adult SHR and Wistar rats, and the S1-BF ROI from this atlas was projected onto co-registered FMISO PET images. As for the FMISO lesion ROI, these results showed no significant effect of hyperoxia on SUV values (data not shown).

Fourth, ischemic cortex PtO_2_ was very low in some SHRs at baseline, and increased only slightly in some rats, which could in part explain the negative findings in SHRs. Liu et al [60] have reported that in rodent MCAo, NBO resulted in marked increases in P_t_O_2_ in the penumbra but not in the ischemic ‘core’, which exhibited very low baseline P_t_O_2_ values similar to that found in some of our SHRs. However, larger increases in P_t_O_2_ might have occurred in other cortical areas that we did not assess, and although it would have been of interest to assess PtO_2_ in additional brain areas, this was not feasible given the available facilities. Furthermore, interpretation of the Liu et al finding [60] is not straightforward, as in severely ischemic conditions the oxygen extraction fraction is near-maximal [4], so that increasing oxygen delivery to viable tissue would result in increased oxygen use and therefore not necessarily in marked increases in P_t_O_2_, despite overall improved oxygenation. One might also argue that such very low P_t_O_2_ values indicate that the affected tissue is already irreversibly damaged (i.e., the ‘core’), and hence that NBO might not have enhanced P_t_O_2_ in such conditions, resulting in unchanged FMISO lesion volumes. However, even though SHRs have worse hypoperfusion [33–35] and hypoxia after MCAo than Wistar rats (as shown here), they do have an extensive penumbra initially [33–35, 62], and early interventions such as reperfusion and hyperoxia but also drugs do salvage the penumbra and reduce final infarct in SHRs [31, 47, 63–66], indicating that NBO should have affected FMISO uptake and retention, albeit probably less so than in Wistar rats.

Fifth, and linked with the point just discussed, although the P_t_O_2_ measurements purposely overlapped most of the FMISO tissue uptake period, allowing their comparison, they began 30mins after MCAo in the ischemic cortex, again to comply with technical constraints (see Methods). We therefore cannot exclude that larger initial increases in P_t_O_2_ (i.e., the first 30 minutes) occurred, and then reduced over time, explaining the lack of changes in the 2-3hrs SUV images used to determine the FMISO lesion volumes. However, our P_t_O_2_ data showed steady increases after hyperoxia until the end of measurement (see Fig 1). Furthermore, because of its trapping process, FMISO SUV measured 2-3hrs after tracer injection reflects uptake from all time points, including early ones [5].

Sixth, again linked with the above two points, FMISO trapping might be sensitive to hyperoxia only for a short duration, while cells are still fully viable. However, in this event inspection of the TACs should have revealed changes in their initial phase, which were not observed (Fig 7), and the kinetic modelling results would also have been affected, including poor fits, which again was not seen.

Seventh, FMISO trapping in hypoxic brain tissue might occur as an all-or-nothing phenomenon, below a P_t_O_2_ higher than that achieved during hyperoxia; however, the previously published relationships between P_t_O_2_ and FMISO trapping in other organs [26, 57], although strongly non-linear, do not support this idea.

Overall, therefore, although the use of small samples precludes us from reaching any definitive conclusion, there are no clear method-related explanations for our unexpected findings. Importantly, however, they are in fact consistent with two previous studies using other nitromidazole derivatives in rodent stroke models, and *ex vivo* instead of *in vivo* imaging here. One 2-hr MCAo study in mice reported no effect of NBO on hypoxic volumes determined *ex vivo* 135min after IV administration of non-radioactive EF-5 [67]; a finding not addressed by the authors. In another 2-hr MCAo study performed in Sprague-Dawley rats, NBO administered together with perfluorocarbons early after MCAo resulted in major (2 to 4-fold) reductions in core volume (assessed using Silver staining) but only minor (15–20%) reductions in hypoxia volume, assessed using the *ex vivo* pimonidazole method 8 or 24hr later [68]. These earlier findings are entirely consistent with our observations. Note that in our study FMISO lesion volumes also slightly decreased under NBO in Wistar rats by ~15% on average (Table 1), although this showed not even a trend for significance due to major individual values overlap (Fig 3).

Thus, an alternative, biochemical hypothesis to explain our negative results is worth considering. For instance, despite higher P_t_O_2_, actual intracellular oxygenation might rapidly decline after a brief initial improvement, due for instance to mitochondrial dysfunction. In favor of this hypothesis, in their report [67] Sun et al found that, 2-hr after start of MCAo, hyperoxia did not significantly affect tissue levels and expression of HIF-1α —a cell protein increased by hypoxia. Effectively, it is known that rather than tissue P_t_O_2_, what nitromidazole derivatives assess is some downstream cell process triggered by hypoxia and involving irreversible trapping into cell organelles, whose intimate mechanisms are still unclear [5, 69].

In our study, practical factors (see Methods) precluded measuring P_t_O_2_ in the same rats that underwent FMISO scans. Assuming these technical issues can be resolved, future FMISO studies should directly compare tracer uptake to brain P_t_O_2_. For similar reasons, laser Doppler blood flow measurements to confirm effective ischemia following MCAo could not be carried out in conjunction with PET. However, we have previously published perfusion values after distal MCAo in SHRs [48], and in addition, all rats in our study had both FMISO and DWI lesions, indicating effective ischemia. To further clarify the relationships between brain P_t_O_2_ and FMISO uptake after MCAo in animal stroke models in both control and NBO conditions, future studies should not only involve at least twice larger samples, but could also map other MR-based tissue markers of ischemia severity, such as ADC and possibly lactate and NAA. These would need to be monitored simultaneously with FMISO PET, which is now feasible using PET/MR hybrid scanners [70]. Likewise, FMISO PET acquisition could run in parallel with pimonidazole brain uptake, allowing hypoxia mapping directly in brain slices collected at the end of PET acquisition; this could also be combined with HIF-1α immunoblotting. Finally, and more generally, biochemistry studies addressing the intricate cellular mechanisms of FMISO trapping in conditions of focal ischemia are warranted.

Turning to the lack of effect of hyperoxia on MRI and TTC lesion volumes in either rat strain, this was not unexpected as it is consistent with the literature mostly indicating no or only marginal reductions in infarct volumes with MCAo durations >2hrs [71–75], including in SHRs [76], in contrast with shorter MCAo [46, 59, 77–83], again including SHRs [31]. Although rodent and clinical studies have documented effective ‘freezing’ of DWI lesion growth during hyperoxia [74, 84, 85], this benefit is lost if reperfusion does not occur soon enough, even despite improved tissue oxygenation. This dependence on early reperfusion is akin that of the penumbra, whereby at some point in time—determined by the severity of hypoperfusion—the penumbra tilts towards irreversible damage [4, 86]. However, why FMISO uptake was not affected by hyperoxia is a different matter, and remains unexplained.

In Wistar rats, MCAo resulted in ~3-fold reductions in ischemic cortex P_t_O_2_ relative to control conditions. Though we could not identify previous similar studies in this strain, this low P_t_O_2_ is broadly consistent with an extensive literature in other rat strains [58–60, 73], mice [46] and non-human primates [87, 88] as well as in man [89–91]. Quite strikingly, ischemic cortex P_t_O_2_ was considerably lower in SHRs than in Wistar rats. We are not aware of previous studies of P_t_O_2_ in SHRs during MCAo. This probably reflects this strain’s poor leptomeningeal collaterals and impaired cerebrovascular autoregulation, resulting from increased vessel stiffness, thicker wall and reduced lumen [39, 41–45].

Normobaric hyperoxia increased ischemic cortex P_t_O_2_ ~2-fold in both strains, consistent with previous literature in Sprague Dawley rats [58, 59] and mice [46]. Thus, the marked (~4-fold) increase in arterial PO_2_ achieved during hyperoxia did improve ischemic tissue oxygenation. Given that persistent tissue hypoxia is the main trigger of the ischemic cascade that eventually leads to irreversible cell death and tissue necrosis [1, 2], these effects of hyperoxia are promising and should foster clinical trials. However, the present study again documents that hyperoxia does not reduce ischemic lesion size if arterial occlusion persists beyond a certain time point.

Another interesting finding from the present study is that baseline cortical P_t_O_2_ was much lower in SHRs as compared to Wistar rats. We are not aware of any previous study of cortical P_t_O_2_ in SHRs. Only Weaver et al [92] reported brain P_t_O_2_ values in adult SHRs, but this was for white matter only, and no data from Wistar rats were presented as control. Interestingly, one study found reduced global cerebral blood flow in stroke-prone SHRs even before the development of infarcts or behavioral impairment [93], thought to indicate the presence of tissue hypoxia. This chronic relative brain hypoxia in SHRs likely relates to the above-mentioned poorer cerebrovascular tree, and likely underlies the much larger effects of MCAo in SHRs discussed above. By extension, chronic brain hypoxia might contribute to the larger ischemic lesions seen in hypertensive patients in the clinical setting [94]. In this respect, it would be interesting to test whether chronic anti-hypertensive therapy increases baseline brain P_t_O_2_ in SHR rats.

*In conclusion*, the unexpected apparent lack of marked effect of hyperoxia on FMISO brain uptake and kinetic rate constants despite partially improved tissue oxygenation observed in this pilot study suggests that in circumstances such as prolonged ischemia, the cellular mechanisms underlying FMISO trapping in hypoxic tissue may be disjointed from interstitial oxygen pressure. A better understanding of FMISO trapping processes will be important for further applications of FMISO PET imaging.
